# Cardioprotective Effects of *Dysphania ambrosioides* Leaves Against Ischemia/Reperfusion‐Induced Myocardial Injury in Langendorff‐Perfused Rat Heart

**DOI:** 10.1155/tswj/2570536

**Published:** 2026-04-27

**Authors:** Mounime Kadi, Ali Berraaouan, Hassane Mekhfi, Abderrahim Ziyyat, Mohamed Bnouham, Abdelkhaleq Legssyer

**Affiliations:** ^1^ Laboratory of Bioresources, Biotechnology, Ethnopharmacology and Health, Biology Department, Faculty of Sciences, Mohammed First University, Oujda, 60000, Morocco

**Keywords:** anti-inflammatory activity, antioxidant activity, cardioprotection, *Dysphania ambrosioides*, infarct size, ischemia/reperfusion

## Abstract

**Background:**

The ethnopharmacological relevance of *Dysphania ambrosioides* (commonly known as M’khinza) is evident in its longstanding traditional use across various cultures, where it is valued for its medicinal properties in addressing a range of health conditions, emphasizing the potential for further pharmacological exploration.

**Objectives:**

The present study aims to investigate the cardioprotection against ischemia–reperfusion (IR) injuries following the administration of aqueous extract (Aq.E) of *D. ambrosioides* leaves in isolated rat hearts according to *Langendorff*.

**Material and Methods:**

The hearts of male Wistar rats were isolated, allowed to acclimate for 30 min, and then experienced global ischemia for 30 min and reperfusion for 120 min. Krebs–Henseleit buffer (KHB) was infused into the control group. However, a solution with 10, 20, and 40 µg/mL of extract was infused into the treatment groups (KHB). The Aq.E anti‐inflammatory, anticoagulant, and antioxidant properties were also assessed, and its mineral composition was examined.

**Results:**

Our results demonstrate that the *D. ambrosioides* leaf Aq.E has considerable cardioprotective activity, evidenced by its beneficial hemodynamic, biochemical, and histological effects in mitigating myocardial lesions induced by the IR sequence.

**Conclusions:**

The findings from this study indicate that the *D. ambrosioides* leaf Aq.E provides cardioprotective benefits in the context of IR injury. This protective effect is attributed to the extract’s antioxidant, anti‐inflammatory, and anticoagulant properties, as evidenced by significant hemodynamic, biochemical, and histological improvements. These findings highlight the potential of *D. ambrosioides* as a medicinal substance for preserving cardiac function and integrity during ischemic events.

## 1. Introduction

All over the world, cardiovascular disorders are considered a leading cause of morbidity and death for both sexes. Among these, ischemia–reperfusion (IR) stands out as a critical clinical model for study due to its significant impact [1]. Redox imbalance, strongly linked to IR, is essential in activating multiple myocardial signaling pathways, primarily driven by reactive oxygen species (ROS) generation [2, 3]. The contribution of these free radicals in triggering and exacerbating the pathogenesis of injuries induced by the IR sequence has been shown by extensive studies [4]. As these lesions become a therapeutic target that requires deep investigations, there is increasing awareness in evaluating the preventive potential of natural antioxidant compounds, which combat oxidative stress and mitigate the damage caused by IR.

The definition of myocardial ischemia is a blood flow decrease to a portion of the myocardium, typically brought about by the partial or total obstruction of one or both coronary arteries [5]. Myocyte cell death is an inevitable consequence of persistent myocardial ischemia without reperfusion [6, 7]. An increasing amount of data indicates that reperfusion, which can be done according to both invasive and noninvasive approaches, is the ultimate solution for ischemic myocardium salvage. However, reperfusion exacerbates the myocardial situation by causing damage in the reperfused tissue due to the massive generation of ROS. The negative effect of free radicals is expressed especially at the level of cardiomyocytes by death via apoptosis [8, 9]. The loss of cardiac cells causes a negative alteration of cardiac performance, via the alteration of hemodynamic, biochemical, and above all histological parameters [10]. In parallel to cardiomyocyte damage, the consequences of IR extend to the level of the coronary network, specifically on the coronary vasculature wall, expressed by the structural deterioration of endothelial cells, which attenuates in advance the endothelium‐dependent cardioprotective effects. These lesions are essentially related to coronary vasoconstriction, platelet adhesion, and aggregation [11]. For this, growing interest has been devoted to the development of ROS scavengers as therapeutic tools that fight against reperfusion injury, in particular, and the IR sequence in general.

In recent years, the use of bioactive nutritional or nutraceutical products has gained increasing scientific interest. It is well known that the modernity of lifestyle has increased the prevalence of cardiovascular risk factors, which increases the chances of potentially fatal morbidity. Therefore, nonpharmacological therapeutic interventions endowed with protective cardiovascular properties could be promising alternatives against cardiovascular pathophysiology [12]. Several clinical and experimental investigations have shown that antioxidants can lower myocardial damage following IR. In this context, there have been reports of several therapeutic plants in the literature, with their cardiovascular benefits largely attributed to their potent antioxidant activities [13].

Eastern Morocco is renowned for its rich biodiversity and abundance of medicinal plants [14]. For this reason, great scientific interest has been given to it in pharmacological studies*. Dysphania ambrosioides* (L) (*D. ambrosioides*), commonly known as M’khinza and extensively grown in subtropical and subtemperate climates, is frequently utilized in traditional medicine. [15–17]. This species, which can be either annual or perennial, is indigenous to Mexico and Central and South America [18]. *D. ambrosioides* has gained significant consideration due to its strong spread in MOROCCO, also due to its antidiabetic [19] and vasorelaxant effects [20]. So far, there is no report on the cardioprotective effects of *Dysphania ambrosioides* aqueous extract (Aq.E) against myocardial damage following IR. Therefore, in the current investigation, the impact of *D. ambrosioides* leaf Aq.E on myocardial damage induced by the IR sequence and on the infarct size was evaluated on the isolated/perfused rat heart according to the Langendorff technique.

## 2. Material and Methods

### 2.1. Preparation of *D. ambrosioides* Leaf Aq.E

*D. ambrosioides* leaves were collected in Oriental Morocco with a voucher example that has been assigned a collection number 4UMPOM 785 in the Department of Biology, Faculty of Sciences (Oujda, MOROCCO). The fresh, mature leaves have been detached from the rest of the plant and allowed to dry at room temperature. 100 g of dry leaves was infused in 1 L of boiling distilled water for 45 min. The crude Aq.E was filtered using adequate filters and dried under lower pressure at 45°C. The extraction yield was 23%. The dried Aq.E was reconstituted using distilled water to have the required doses for the rest of the experiment.

### 2.2. Mineral Content of *D. ambrosioides* Leaf Aq.E

The X‐ray fluorescence energy‐dispersive spectroscopy (EDX‐7000, C142‐E037A, Shimadzu) instrument was used to determine the mineral content of *D. ambrosioides* leaf Aq.E according to Ref. [21]. High‐energy X‐rays are used by X‐ray fluorescence spectrometers to excite fluorescent radiation from biological samples to perform elemental analysis. This analysis is endowed with great precision and rapidity. When an X‐ray tube irradiates the sample, fluorescent X‐rays are produced by its atoms, with a wavelength and energy characteristic of the element producing them, whose X‐ray intensity is a function of its concentration in the crude Aq.E. Thus, mineral composition was quantified from fluorescence intensities and expressed as relative weight percentages (% w/w), normalized to the total dry weight of the crude Aq.E.

### 2.3. Animal Housing

Isolated rat hearts were prepared from normotensive Wistar rats (assessed using tail‐cuff plethysmography) aged between 2 and 3 months (240–280 g), were fed and given water ad libitum, and were maintained under standard laboratory conditions (light/dark cycle of 12h/12 h and a temperature of 24°C ± 2°C) in a well‐ventilated space. The use of rats weighing 240–280 g in our IR model was carefully chosen to enhance the clinical relevance of our findings, align with established research practices, and provide consistent and reproducible results. We believe that this choice strengthens the overall validity and applicability of our study to human cardiovascular disease. The study was carried out in accordance with the globally recognized norms for the use and care of laboratory animals as stated in the European Community guidelines (EEC Directive of 1986; 86/609/EEC) or the US guidelines (NIH publication #85‐23, revised in 1985). The animal experiments conducted in this study were approved by the Institutional Ethics Committee of the Faculty of Sciences, Oujda, under the approval number 02/26‐LBBEH‐01.

### 2.4. Modified Langendorff Isolated Perfused Heart Preparation

Male Albino Wistar rats (240–280 g) were put under anesthesia with sodium pentobarbital (40 mg/kg intraperitoneal injection (i.p.)). Upon loss of pedal and tail reflexes, thoracic surgery was performed to rapidly excise the heart. The beating heart was then submerged in a cold perfusion buffer and immediately perfused via the aorta according to *Langendorff*’s technique at a steady rate (12 mL/min) with the following Krebs–Henseleit buffer (KHB) composition (mM): NaCl, 118; KCl, 4.7; MgSO_4_, 1.2; KH_2_PO_4_, 1.2; NaHCO_3_, 25; glucose, 11; Na‐pyruvate, 2; CaCl_2_, 1.8. The buffer was constantly bubbled and filtered with a mixture of 5% carbon dioxide and 95% oxygen to maintain a pH = 7,35. Both the heart bath and the perfusate were retained at a constant temperature (37°C) and continuously monitored [22].

### 2.5. Experimental Protocol: Myocardial IR Model

Thirty male Wistar rats were randomly allocated into five experimental groups (*n* = 6 per group) to evaluate the cardioprotective effect of *D. ambrosioides* leaf Aq.E against myocardial IR injuries. Hearts were mounted on a Langendorff apparatus and perfused with KHB at a constant flow rate of 12 mL/min. All hearts have an initial 30‐min equilibration period. In the control (IR) group, KHB was used to perfuse hearts during the experiment and experienced 30‐min global isothermal ischemia (oxygenated KHB delivery to the entire myocardium is completely restricted, and the heart was maintained at a constant temperature), followed by 120‐min reperfusion [23]. For verapamil (reference cardioporotective drug through calcium channel blockade) and *D. ambrosioides* leaf Aq.E, hearts were perfused with verapamil (10 µg/L) [24] or Aq.E (10, 20, and 40 µg/mL), those treatments were introduced during the last 5 min of the equilibration period before ischemia and were continuously perfused throughout the entire 120‐min reperfusion period [23].

### 2.6. Measurements of Hemodynamic Functional Recovery

For the purpose of analyzing hemodynamic data, a degassed water‐latex‐filled balloon connected to a pressure transducer was inserted inside the left ventricle through the mitral valve. The latex balloon was inflated until LVEDP reached a range of 4–10 mmHg with a volume of 120–130 µL held constant throughout the experiment. A catheter attached to the aortic perfusion cannula was used for the coronary perfusion pressure (CPP) recording. Functional recovery on reperfusion was assessed by measuring left ventricular end‐diastolic pressure (LVEDP), left ventricular developed pressure (LVDP), arrhythmia, CPP, time derivatives of the pressure changes during contraction (+dP/dt), and time derivatives of the pressure changes during relaxation (−dP/dt) [25].

### 2.7. Anti‐Inflammatory Effect of *D. ambrosioides* Leaf Aq. E: Membrane Lysis Assay

#### 2.7.1. Protein Denaturation Assay

The protein denaturation assay was carried out following the protocol [26] with some modifications. A 0.2% (w/v) bovine serum albumin (BSA) solution was prepared in a tris‐saline buffer solution (glacial acetic acid was used to bring the pH down to 6.8). For the test group, a number of concentrations must be prepared of *D. ambrosioides* Aq.E using ethanol as solvent. Five milliliters of 0.2% BSA was produced in test tubes, to which 0.05 mL of each concentration of the test group was added. A range of varying concentrations (100–250 µg/mL) of ibuprofen (anti‐inflammatory of reference) was prepared and served as a standard group, to which 5 mL of 0.2% BSA was added. Test tubes were subjected to thermal stress (75°C/5 min), then left to cool. The determination of the absorbance of the solutions was carried out at 660 nm using a UV‐visible spectrophotometer. All tests were carried out in triplets, and the averages were calculated.

The percentage % of inhibition of BSA protein denaturation was determined using the following formula:

(1) %inhibition of BSA denaturation=optical density OD control−OD testOD control×100.

*OD* control corresponds to the measurement of the heated solution OD.

The *OD* test is the measurement of the heated test solution OD.

#### 2.7.2. Red Blood Cell (RBC) Membrane Stabilization Test

The anti‐inflammatory activity of *D. ambrosioides* Aq.E was assessed using the RBC technique [27, 28]. The protocol followed was taken from the reported literature with some modifications. Wistar rats weighing 240–280 g that had not been given any NSAIDs for two weeks prior to the experiment were put to sleep with ether. Fresh blood was drawn from the abdominal aorta via catheterization and placed in glass tubes with equal parts of the sterile anticoagulant solution (2% dextrose, 0.8% sodium citrate, 0.5% citric acid, and 0.42% sodium chloride). Cell pellet volumes were measured, erythrocyte cell concentrates were centrifuged at 3000 rpm for 15 min, and then rinsed with sterile saline (0.9% w/v NaCl, pH 7.2) until the supernatant turned clear. A 10% suspension was then reconstituted using a sterile solution of sodium phosphate buffer (adjusted pH 7.4) at 10 mM containing: 0.2 g NaH2PO4, 1.15 g Na2HPO4, and 9 g NaCl for a volume of 1 L of distilled water. The reconstituted RBCs were used as such for the heat or hypotonicity (H/H)‐induced hemolysis tests. The hemolysis percentage was estimated assuming that the hemolysis produced in the control was 100%.

H/H stress‐induced hemolysis of rat RBCs was used to assess the stabilizing effect of *D. ambrosioides* Aq.E on the erythrocyte membrane.

##### 2.7.2.1. Hemolysis Induced by Erythrocyte Hypotonicity

0.1 mL of the 10% erythrocyte suspension was added to distilled water to create a final volume of 5.1 mL, which was used to manufacture various concentrations of *D. ambrosioides* Aq.E. Diclofenac sodium served as the anti‐inflammatory medication of reference. After an hour of incubation at 37°C, the reaction mixtures were centrifuged for 15 min at 3500 rpm. A spectrophotometer set to 540 nm was used to measure the amount of hemoglobin in the supernatant; the experiment was run in triplets, and the mean values were computed.

The percentage of hemolysis caused by erythrocyte hypotonicity that was inhibited was calculated using the following expression [27]:

(2) %inhibition of hemolysis induced by erythrocyte hypotonicity=OD control−OD testOD control×100.

The OD test measures the OD of the sample being tested in the hypotonic solution, whereas the OD control measures the OD of the hypotonic control.

##### 2.7.2.2. Hemolysis Induced by Erythrocyte Heat

The isotonic phosphate‐buffered solution (0.15 M, pH 7.4) was used to prepare various concentrations of *D. ambrosioides* Aq.E. 0.1 mL of the 10% erythrocyte suspension was added to the final amount of 5.1 mL. The buffer was used to construct a standard tube with 5 mL of Diclofenac sodium (a reference anti‐inflammatory medicine) at several doses and a control with 5 mL of the vehicle solution (isotonic phosphate buffer). The tubes were then allowed to cool to room temperature after all of the reaction mixtures had been incubated for 30 min at 60°C. Lastly, centrifugation was carried out for 10 min at 3000 rpm. A spectrophotometer set to 540 nm was used to measure the amount of hemoglobin in the supernatant solution. Triplets are used for the tests, and the mean scores have been determined.

To calculate the percentage inhibition of hemolysis caused by erythrocyte heat, the following expression was utilized [28]:

(3) %inhibition of hemolysis induced by erythrocyte heat=OD control−OD testD control×100.

The OD test measures the OD of the heated sample solution, whereas the OD control measures the OD of the heated control solution.

### 2.8. Anticoagulant Activity of *D. ambrosioides* Leaf Aq.E

#### 2.8.1. Bleeding Time (BT)

The effect of *D. ambrosioides* Aq.E on BT was evaluated using the tail section technique [29] with some modifications. Five groups of albino mice weighing 20–30 g were randomly assigned (mice are more suitable than rats for small‐volume blood collection), the test group received different concentrations of the plant Aq.E (10, 20, and 40 mg/kg body weight [BW]), the control group was given distilled water (1 mL/100 g BW), and the last group received acetylsalicylic acid (ASA: 30 mg/kg BW) as the reference drug (all drugs were administered by single gavage). Two hours later, the mice were anesthetized using sodium pentobarbital (50 mg/kg i.p.), then placed on a hotplate, and the body was thermostated (37°C). A sterile razor blade was used to sanctify a distal segment of the tail (1.5 cm). Every 5 seconds, blood was dabbed onto filter paper until the stain disappeared. The amount of time needed for the bleeding to halt after section was known as the BT. Triplets were used for all studies, and mean values were computed.

#### 2.8.2. Plasma Recalcification Time (PRT) Assay

The anticoagulant activity of *D. ambrosioides* Aq.E was assessed by the determination of PRT reported in the literature with some modifications [30]. Wistar rats (250–280 g) were anesthetized using sodium pentobarbital (50 mg/kg i.p.), and fresh blood was drawn from the abdominal aorta in tubes containing ethylenediaminetetraacetic acid (EDTA, 0.1 M). Plasma was separated by centrifugation (1500 rpm/10 min) and maintained at 37°C (100 µL). The common coagulation pathway was triggered by adding 200 µL calcium chloride (0.15 M). The time interval required for plasma recalcification was noted within 10 min after intravenous injection through the jugular vein of *D. ambrosioides* Aq.E at different concentrations (10, 20, and 40 mg/kg) and compared with the reference anticoagulant drug (heparin 1 mg/kg). Distilled water was used for the control group. Three duplicates of each experiment were conducted, and mean values were calculated.

### 2.9. Antioxidant Enzyme Assays

#### 2.9.1. Preparation of Tissue Homogenate

For cardiac homogenate preparation, a phosphate buffer solution is prepared (10 mL, pH 7.4) for cardiac tissue grinding. Afterward, centrifugation at 8000 g for 15 min at 4°C was carried out. The tissue homogenate supernatants were collected and stored for subsequent analyses. The determination of protein concentration and enzyme activity assays was performed as described below.

##### 2.9.1.1. Myocardial Superoxide Dismutase (SOD) Activity

The determination of the superoxide scavenging activity of SOD was performed by measuring the inhibitory activity of the reduction of nitroblue tetrazolium (NBT) to blue‐colored formazan at 560 nm by the superoxide anions generated through hydroxylamine hydrochloride photo‐oxidation. The SOD enzyme activity was expressed in U/mg of protein [31].

##### 2.9.1.2. Myocardial Catalase (CAT) Activity

CAT enzyme activity was measured based on the rate of disappearance of hydrogen peroxide (H2O2) intensity at 240 nm through a UV spectrophotometric method as described by [32]. The rate of OD change was recorded, and the CAT activity was expressed in U/mg protein. One unit (U) is defined as the amount of CAT required to degrade 1 μmol of H_2_O_2_ per minute at 25°C and pH 7.

##### 2.9.1.3. Myocardial Hydrogen Peroxide Activity (H_2_O_2_)

The determination of H_2_O_2_ content in myocardial tissue was done according to [33] with some modifications.

##### 2.9.1.4. Assessment of Myocardial Lipid Peroxidation (Measured by Malondialdehyde [MDA] Levels)

Lipid peroxidation in the cardiac homogenate was determined by measuring MDA activity through a spectrophotometric method with some modifications [34].

##### 2.9.1.5. Myocardial Protein Estimation

Myocardial protein content was determined using the Bradford colorimetric assay, based on Coomassie Brilliant Blue dye binding to proteins, with BSA used as the standard for calibration [35].

### 2.10. Determination of Myocardial Infarct Size

After 120 min of reperfusion, the hearts of rats were promptly taken out of the Langendorff equipment for the determination of the infarct size according to the NBT staining technique with minor modifications [36]. Briefly, the hearts were incubated with NBT at 0.25 w/v (0,1 M phosphate buffer) after being sliced into transverse slices that were 2 mm thick, avoiding light for 15/20 min at 37°C with rotation every 2 min to allow uniform and complete tissue staining. A sufficient volume of incubation solution was used to completely cover the heart slices. Using ImageJ software, computerized planimetry was used to calculate the size of the infarcted area (in cm2), and then, it was cut and weighed. The infarct size ratio was expressed as [infarct area]/[total heart slices area].

### 2.11. Data Analysis

The results of experiments were presented as mean ± S.E.M. The statistical analyses were performed using GraphPad Prism, Version 8.0.2. Significance was accepted when *p*‐value is under < 0.05.

## 3. Results

### 3.1. Elemental Concentration of *D. ambrosioides* Leaf Aq.E

**FIGURE 1:**
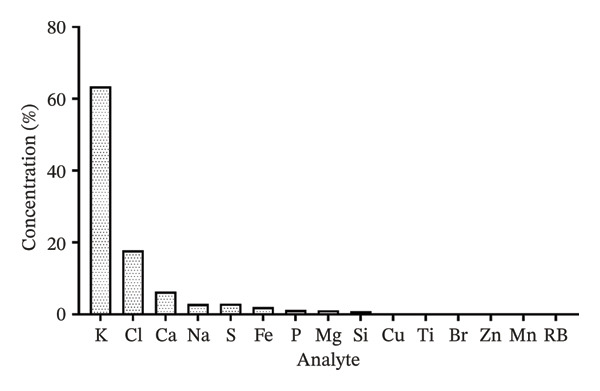
Mineral content in (%) of *D. ambrosioides* leaf Aq.E. No standard error bars are shown, as EDX measurements were performed as a single determination for each sample.

**FIGURE 2:**
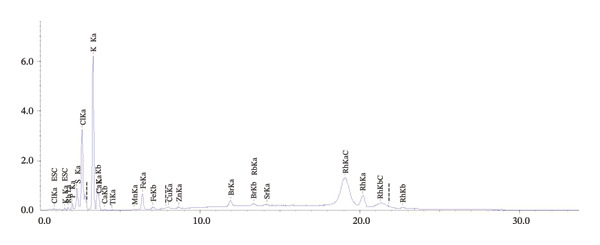
Original software‐generated graphic of the elemental peaks.

Using the X‐ray fluorescence EDX‐7000 instrument (Figures 1 and 2), it was found that potassium (K) is the most abundant macromolecule in *D. ambrosioides* leaf Aq.E, presenting a concentration of 63.463 ± 1.45%, followed by chlorine (Cl) (17.8575 ± 0.003%), and then calcium (Ca) (6.347 ± 1.13%).

### 3.2. Changes in Hemodynamic Parameters Following IR Injury

To analyze whether *D. ambrosioides* leaf Aq.E was able to maintain heart function, hemodynamic metrics such as LVEDP (Figure 3(a)), LVDP (Figure 3(b)), CPP (Figure 3(c)), +dP/dt−dP/dt (Figure 3(d)), and arrhythmia (Figure 3(e)) were measured. There was a significant decrease in LVEDP (57.82 ± 10.30 mmHg, *p* < 0.01) and CPP (90.19 ± 0.89 mmHg, *p* < 0.001) in verapamil pretreated groups compared with IR (101.14 ± 7.33 mmHg, 101.49 ± 0.71 mmHg, respectively). For Arrhythmia %, there was also a significant decrease (18.96 ± 12.41%, *p* < 0.001) in verapamil pretreated groups compared with IR (96.21 ± 1.15%). Cardiac LVEDP, CPP, and Arrhythmia % remained significantly lowered in *D. ambrosioides* leaf Aq.E lower, medium, and higher doses compared with the IR group. For the cardiac LVDP, there was a significant increase (84.40 ± 1.24 mmHg, *p* < 0.001) in groups pretreated with verapamil compared with IR (15.44 ± 2.12 mmHg). The Aq.E was also capable of improving LVDP in a dose‐dependent manner up to 101.77 ± 6.09 mmHg for 40 µg/mL. For time derivatives of the pressure changes during contraction (+dP/dt) and relaxation (−dP/dt), it was found that verapamil significantly increased (*p* < 0.01) the levels of +dP/dt (3228.81 ± 25.11 mmHg/s) and −dP/dt (2749.03 ± 48.93 mmHg/s). *D. ambrosioides* leaf Aq.E also improved in a dose‐dependent manner and significantly + dP/dt and −dP/dt levels compared with the IR group.

FIGURE 3Effect of *D. ambrosioides* leaf Aq.E and verapamil on (a) cardiac LVEDP (mm·Hg), (b) LVDP (mm·Hg), (c) CPP (mm Hg), (d) (±) dP/dT (mm·Hg), and (e) arrhythmia (%) post‐IR. Repeated measurements were performed, ^∗^ *p* < 0.05, ^∗∗^ *p* < 0.01, ^∗∗∗^ *p* < 0.001 compared to IR group, ^#^ *p* < 0.05, ^##^ *p* < 0.01, ^###^ *p* < 0.001 compared to the verapamil group.

**Figure:**
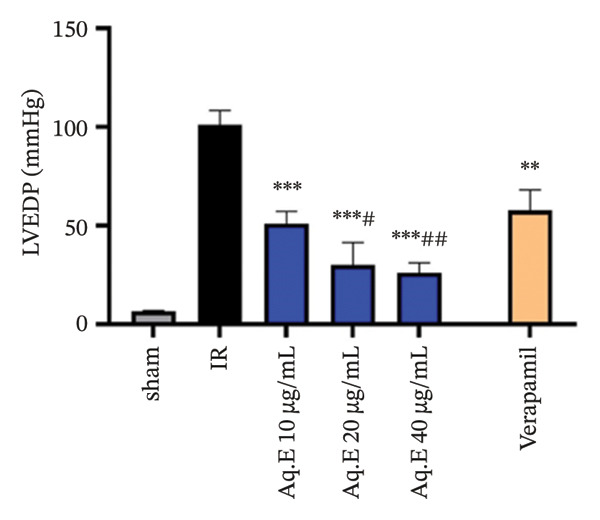
(a)

**Figure:**
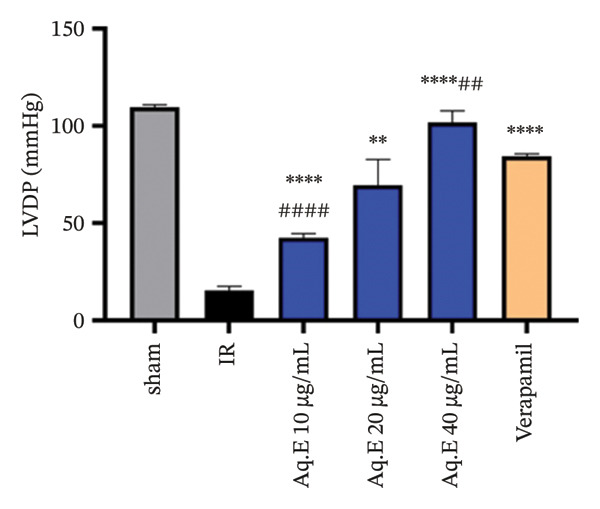
(b)

**Figure:**
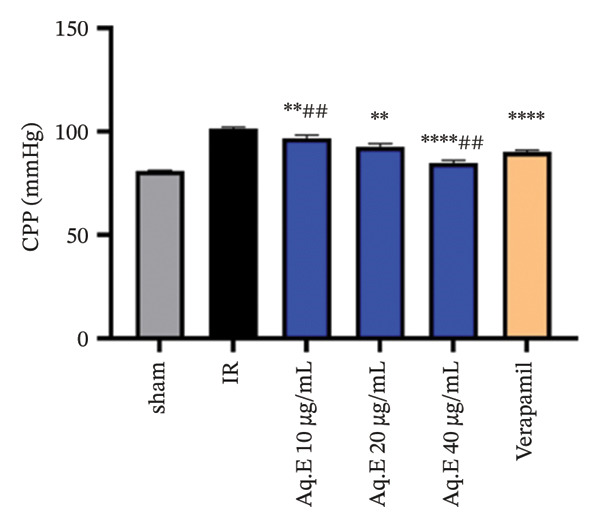
(c)

**Figure:**
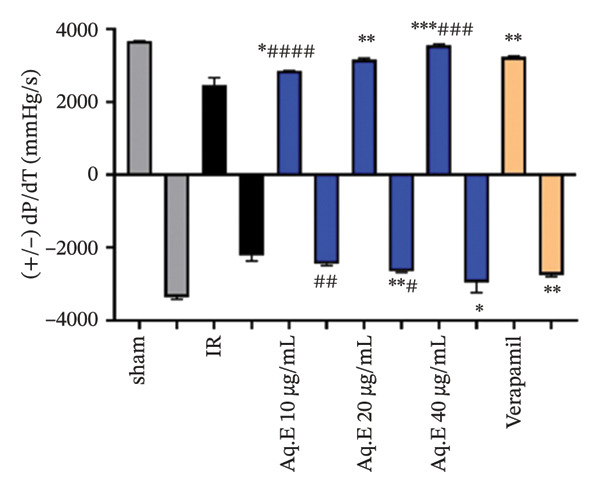
(d)

**Figure:**
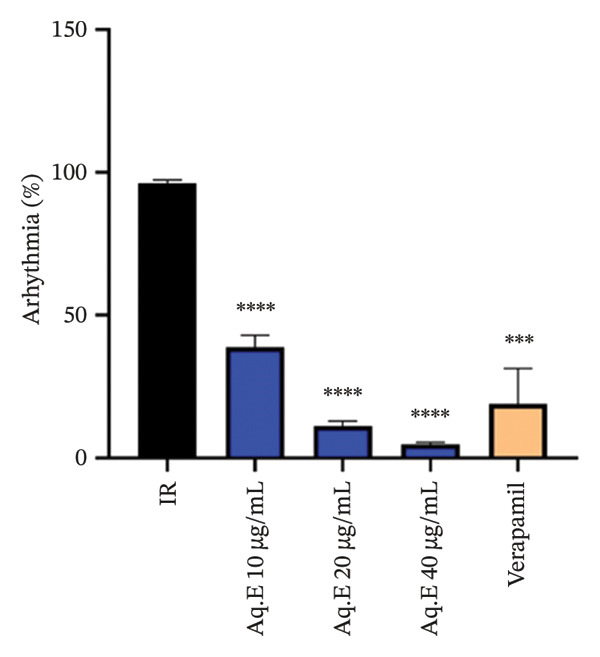
(e)

### 3.3. Anti‐Inflammatory Effect of *D. ambrosioides* Leaf Aq.E

#### 3.3.1. Assay for Protein Denaturation

**FIGURE 4:**
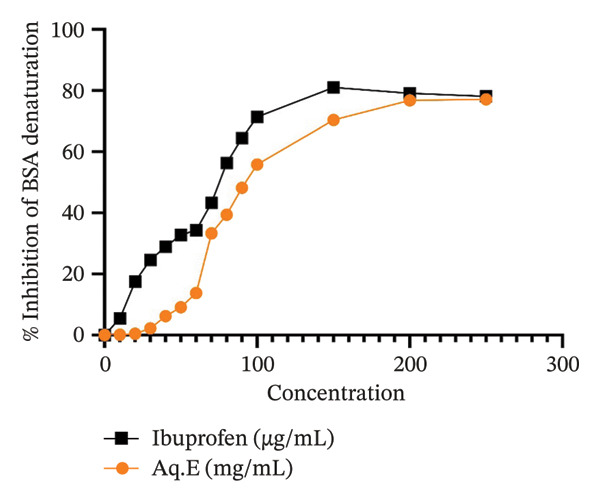
% Inhibition of *D. ambrosioides* leaf Aq.E against BSA protein denaturation in comparison with Ibuprofen.

*D. ambrosioides* leaf Aq.E (half maximal inhibitory concentration IC50 = 95.91 mg/mL) exhibits a possible dose‐dependent inhibitory effect against BSA denaturation, per the recorded results (Figure 4). In contrast, ibuprofen’s inhibitory activity (IC50 = 72.33 µg/mL) demonstrated a maximum inhibitory impact of 81.05% at an actual concentration of 150 µg/mL, albeit comparatively weaker.

#### 3.3.2. Assay for RBC Membrane Stabilization

**FIGURE 5:**
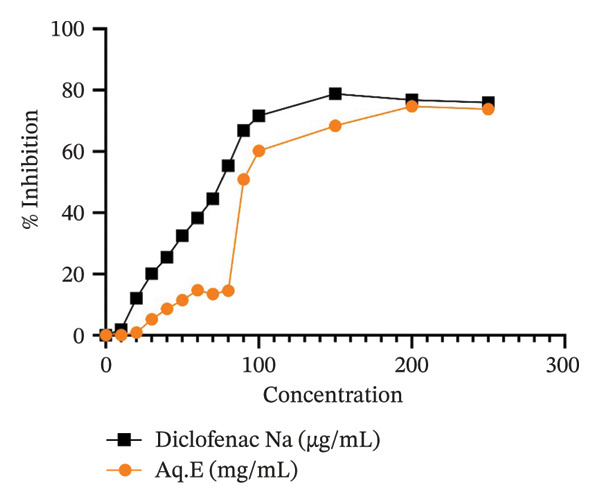
% Inhibition of *D. ambrosioides* leaf Aq.E against heat‐induced RBC denaturation in comparison with Diclofenac Na.

**FIGURE 6:**
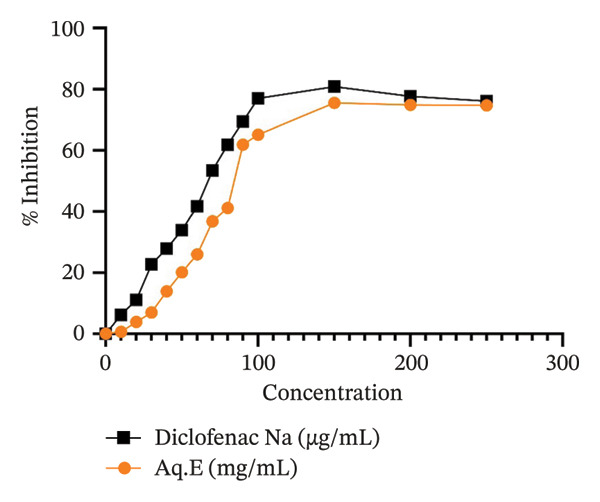
% Inhibition of *D. ambrosioides* leaf Aq.E against hypotonia‐induced RBC denaturation in comparison with Diclofenac Na.

The H/H‐induced hemolysis was effectively inhibited by Aq.Es of the *D. ambrosioides* leaves under study (Figures 5 and 6, respectively). The findings demonstrated protection against rat RBC hemolysis that was dose‐dependent. *D. ambrosioides* Aq.E. showed the maximum percentage of activity against rat RBC hemolysis generated by H/H, respectively, at 200 and 150 mg/mL. Aq.E. reduced the heat‐induced hemolysis with an IC50 of 103.02 mg/mL (IC50 of Diclofenac Na = 72.48 µg/mL). Conversely, Aq.E demonstrated an IC50 of 85.34 mg/mL for the suppression of hemolysis caused by hypotonicity (Diclofenac Na IC50 of 66.70 µg/mL).

### 3.4. Anticoagulant Activity Effect of *D. ambrosioides* Leaf Aq.E

#### 3.4.1. BT

The control group had a BT of 3.39 ± 0.15 min, and the positive control group (ASA) was able to significantly extend the bleeding period of the mouse tail vein to 6.66 ± 0.30 min (*p* < 0.001) compared with the control group. Likewise, *D. ambrosioides* leaf Aq.E may cause a significant dose‐dependent increase in BT (3.62 ± 0.04 min, *p* < 0.01, and 3.87 ± 0.05 min, *p* < 0.01, respectively, at 10 and 20 mg/kg) in comparison with the control group. It may cause a significantly longer BT than ASA (7.16 ± 0.21 min; *p* < 0.001 in comparison with the control group) at the maximum dose of 40 mg/kg. However, when administered to mice, it did not cause any myotoxicity or death.

#### 3.4.2. PRT

Using healthy rat plasma, the recalcification time (RT) assay was used to evaluate the *in vitro* anticoagulant qualities of *D. ambrosioides* leaf Aq.E. The observed normal PRT was 1.16 ± 0.06 min. Heparin administration as a positive control may cause the time to be delayed to 4.64 ± 0.03 min (*p* < 0.001). *D. ambrosioides* leaf Aq.E. caused a significant dose‐dependent delay in time, giving a value of 1.34 ± 0.05, *p* < 0.001, and 3.51 ± 0.05, *p* < 0.001, respectively, at 10 and 20 mg/kg in comparison with the control group, reaching 4.63 ± 0.04 min (*p* < 0.001) at 40 mg/kg. This medication is either less or equally effective than heparin.

### 3.5. Alterations in Endogenous Antioxidants and Cardiac Lipid Peroxidation After IR Injury

In our study, we focused on determining the levels of SOD, CAT, H2O2, and MDA specifically after the reperfusion phase to evaluate the oxidative stress response following IR injury. Assessing these markers at baseline (after 30 min of stabilization) would have required additional experimental groups and, consequently, an increased number of animals, raising ethical considerations. This approach justifies the absence of normal baseline levels for individual markers studied.

#### 3.5.1. Myocardial SOD Levels

**FIGURE 7:**
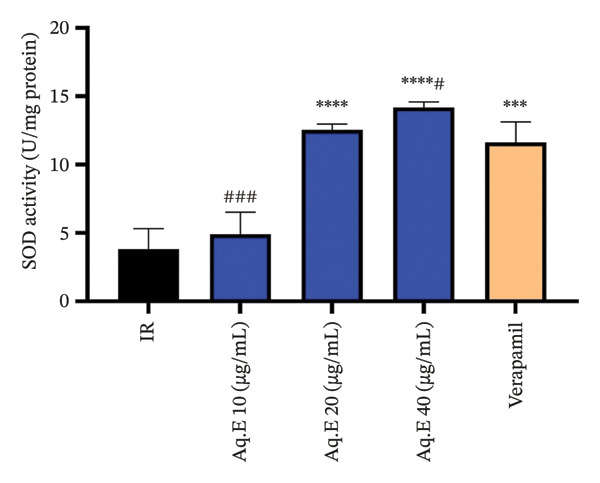
Effect of *D. ambrosioides* leaf Aq.E and verapamil on myocardial SOD activity (U/mg protein) post‐IR. Repeated measurements were performed, ^∗^ *p* < 0.05, ^∗∗^ *p* < 0.01, ^∗∗∗^ *p* < 0.001 compared to the IR group, ^#^ *p* < 0.05, ^##^ *p* < 0.01, ^###^ *p* < 0.001 compared to the verapamil group.

As shown in Figure 7, there was a significant (*p* < 0.001) increase in myocardial SOD activity (11.63 ± 1.49 U/mg protein) in the IR groups pretreated with verapamil in comparison with the IR group (3.82 ± 1.5 U/mg protein). Myocardial SOD activity remained significantly higher in *D. ambrosioides* medium‐dose IR (12.54 ± 0.42 U/mg protein, *p* < 0.001) and higher dose IR (14.18 ± 0.39 U/mg protein, *p* < 0.001) groups in comparison with the IR group.

#### 3.5.2. Myocardial CAT Levels

**FIGURE 8:**
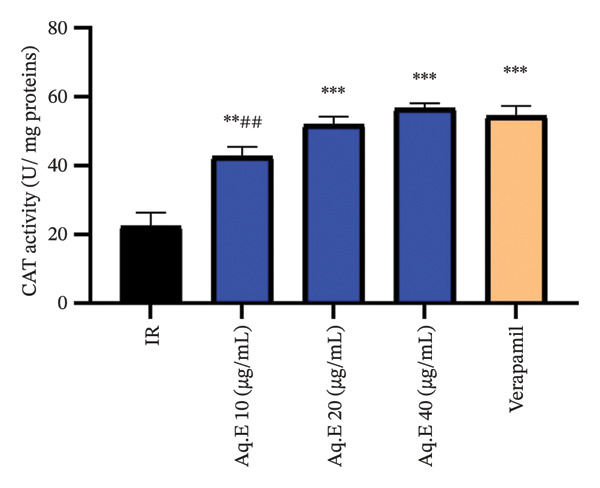
Effect of *D. ambrosioides* leaf Aq.E and verapamil on myocardial CAT activity (U/mg protein) post‐IR. Repeated measurements were performed, ^∗^ *p* < 0.05, ^∗∗^ *p* < 0.01, ^∗∗∗^ *p* < 0.001 compared to the IR group, ^#^ *p* < 0.05, ^##^ *p* < 0.01, ^###^ *p* < 0.001 compared to the verapamil group.

From Figure 8, there was a significant (*p* < 0.001) increase in myocardial CAT activity (54.46 ± 2.88 U/mg protein) in IR groups pretreated with verapamil in comparison with the IR group (22.38 ± 3.97 U/mg protein). Myocardial CAT activities remained significantly higher in *D. ambrosioides* lower dose (42.67 ± 2.77 U/mg protein, *p* < 0.01), medium‐dose IR (51.48 ± 2.27 U/mg protein, *p* < 0.001), and higher dose IR (58.38 ± 2.78 U/mg protein, *p* < 0.001) groups in comparison with the IR group.

#### 3.5.3. Myocardial H_2_O_2_ Levels

**FIGURE 9:**
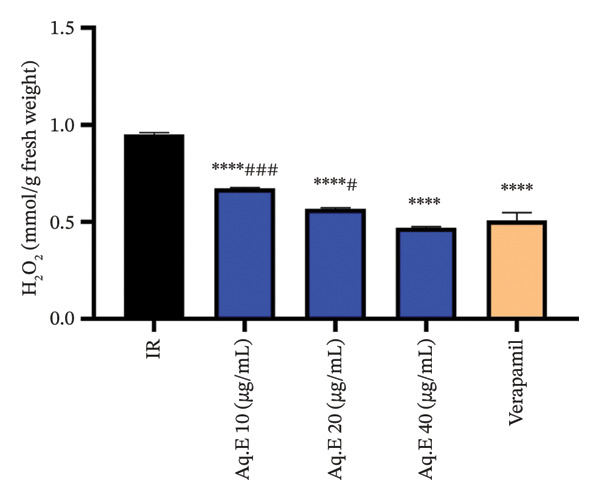
Effect of *D. ambrosioides* leaf Aq.E and verapamil on myocardial H_2_O_2_ activity (mmol/g fresh weight) post‐IR. Repeated measurements were performed, ^∗^ *p* < 0.05, ^∗∗^ *p* < 0.01, ^∗∗∗^ *p* < 0.001 compared to the IR group, ^#^ *p* < 0.05, ^##^ *p* < 0.01, ^###^ *p* < 0.001 compared to the verapamil group.

There was a significant (*p* < 0.001) decrease in myocardial H_2_O_2_ level in verapamil IR groups (0.5 ± 0.04 mmol/g fresh weight) in comparison with the IR group (0.95 ± 0.009 mmol/g fresh weight). Myocardial MDA level remained significantly (*p* < 0.001) lowered in IR *D. ambrosioides* lower dose (0.67 ± 0.005 mmol/g fresh weight), medium‐dose IR (0.56 ± 0.005 mmol/g fresh weight), and higher dose IR groups (0.47 ± 0.004 mmol/g fresh weight) compared to the IR group (Figure 9).

#### 3.5.4. Myocardial MDA Levels

**FIGURE 10:**
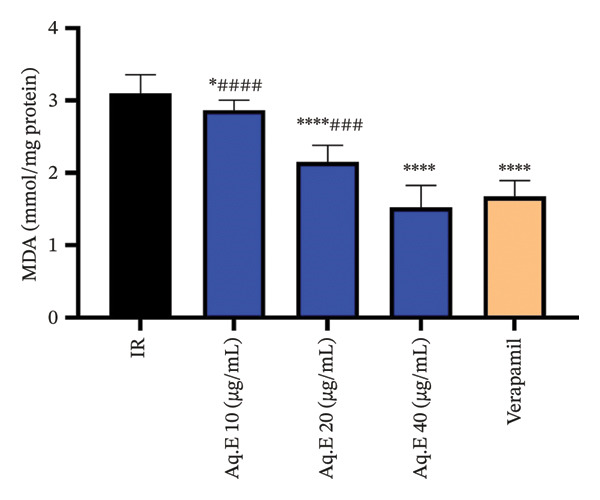
Effect of *D. ambrosioides* leaf Aq.E and verapamil on myocardial MDA activity (mmol/mg protein) post‐IR. Repeated measurements were performed, ^∗^ *p* < 0.05, ^∗∗^ *p* < 0.01, ^∗∗∗^ *p* < 0.001 compared to the IR group, ^#^ *p* < 0.05, ^##^ *p* < 0.01, ^###^ *p* < 0.001 compared to the verapamil group.

There was a significant (*p* < 0.001) decrease in myocardial MDA level in verapamil IR groups (1.67 ± 0.22 mmol/mg protein) compared with the IR group (3.1 ± 0.25 mmol/mg protein). Myocardial MDA level remained significantly lowered in IR *D. ambrosioides* lower dose (2.86 ± 0.13 mmol/mg protein, *p* < 0.05), medium‐dose IR (2.15 ± 0.22 mmol/mg protein, *p* < 0.001), and higher dose IR groups (1.52 ± 0.3 mmol/mg protein, *p* < 0.001) compared to the IR group (Figure 10).

### 3.6. Myocardial Infarct Size Determination

ImageJ applies specific color thresholds to distinguish the infarcted (paler) myocardial tissue from viable tissue (dark red). This segmentation isolates areas of interest, such as the infarcted region, by color differences. Once segmented, ImageJ calculates the infarcted myocardium area (highlighted in yellow), based on pixel count. Then, ImageJ uses the ratio of the infarcted area to the total myocardial area to calculate infarct size as a percentage, providing a precise quantification of the damaged region (Table 1).

**TABLE 1:** Quantitative analysis of myocardial infarct size in heart slices: original and ImageJ‐processed images.

	**IR**	**Aq.E 10 µg/mL**	**Aq.E 20 µg/mL**	**Aq.E 40 µg/mL**	**Verapamil**

Heart slice before image treatment					

Myocardial infarct size measurement					

**FIGURE 11:**
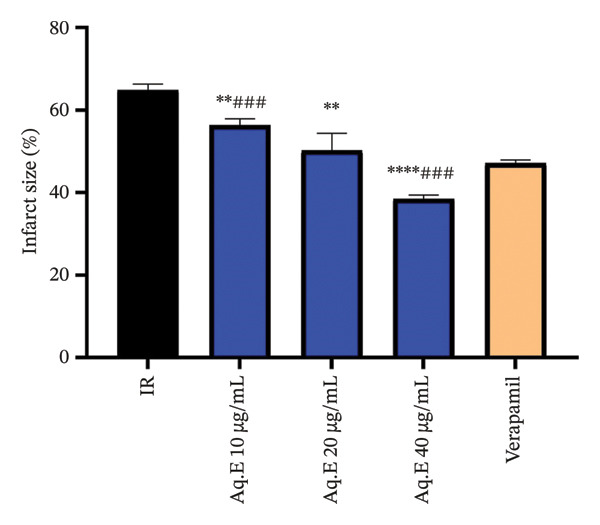
Effect of *D. ambrosioides* leaf Aq.E and verapamil on myocardial Infarct size (%) post‐IR. Repeated measurements were performed, ^∗^ *p* < 0.05, ^∗∗^ *p* < 0.01, ^∗∗∗^ *p* < 0.001 compared to the IR group, ^#^ *p* < 0.05, ^##^ *p* < 0.01, ^###^ *p* < 0.001 compared to the verapamil group.

Based on the examination of NBT staining (Figure 11, Table 1), the infarct size presented in the IR rat hearts is 64.91 ± 1.42%. Pretreatment with *D. ambrosioides* leaf Aq.E (10, 20, and 40 µg/mL) resulted in dose‐dependent reduction in the infarct size, 56.36 ± 1.56%, 50.27 ± 4.12%, and 38.51 ± 0.89%, respectively, while the infarcted area in verapamil pretreated groups was 47.17 ± 0.74%.

## 4. Discussion

### 4.1. For the Mineral Content of *D. ambrosioides* Leaf Aq.E

Mineral elements in plants are essentially classified into microelements and macroelements, presenting important biological roles in the cell. The elemental composition of *D. ambrosioides* is shown in Figure 1. Our results clearly indicate that Potassium K+ is the most prevalent macroelement in *D. ambrosioides* leaf Aq.E (63% and 46%). A vital component of preserving cardiovascular homeostasis is potassium, which is discovered to be actively involved in a number of processes, including the activation of enzymes [37, 38]. Many studies have shown its importance in protecting against cardiovascular damage after increased generation of ROS [39]. Potassium state maintenance is considered among the new emerging cardioprotective therapies [40]. Many experimental studies have shown that potassium supplementation is protective of vascular and tissue biology [41]. Several studies on animal models have shown the inhibitory effect of potassium against ROS generation, vascular smooth muscle cell proliferation, platelet aggregation, and arterial thrombosis by reducing the cholesterol concentration in the vascular wall [42, 43]. Other experimental data in the same process have shown that potassium supplementation plays an essential role against intima thickening and, therefore, against endothelial dysfunction induced by salt, by attenuating NADPH oxidase activity [44, 45]. In the heart, potassium is among the most important electrolytes in the cardiomyocyte that helps to regulate heartbeat [46]. Except during myocardial ischemia, the most lost electrolyte by the cell is potassium [47]. It is well known that potassium supplementation enhances antioxidant enzyme activities such as for CAT and SOD [48]. Additionally, it has a protective impact on the left ventricle’s active relaxation, achieved in part via the suppression of NADPH oxidase activity in the heart, which in turn has a major role as an ROS producer in the heart of patients with ischemic cardiomyopathy [49]. In addition, it should be noted that an improvement in cardiac hypertrophy was observed by potassium supplementation [50]. These very interesting biological effects of potassium can explain both the preventive and cardioprotective effects of *D. ambrosioides* leaf Aq.E, first against endothelial dysfunction, and coronary thrombosis formation. Also, these effects may contribute to the recovery of hemodynamic parameters and improvement of antioxidant status in ischemic myocardium. Hence, sufficient potassium supplementation through pretreatment with *D. ambrosioides* leaves could be considered an interesting strategy for cardioprotection.

### 4.2. For the Anti‐Inflammatory and Anticoagulant Activity of *D. ambrosioides* Leaf Aq.E

The anti‐inflammatory property of *D. ambrosioides* leaf Aq.E has been studied through the BSA protein denaturation caused by heat assay. Protein denaturation is among the principal causes of the inflammatory process, which is well documented in the literature [51, 52], in which the secondary and tertiary protein structures are altered. The molecular mechanism of protein alteration is essentially based on the deterioration of hydrogen, hydrophobic, electrostatic, and disulfide bonds, which leads to the loss of biological function of proteins [53]. Since ROS can directly damage membranes and proteins during myocardial ischemia, it is well recognized that it plays a crucial role in the development of myocardial IR injury [54]. Studies have demonstrated that ROS accumulation triggers myocardial reperfusion injury associated with inflammation [55]. Thus, ROS causes Ca2+ reentry, NO production, platelet activation, metabolic alterations, endothelial dysfunction, and infiltration by inflammatory cells during myocardial ischemia [56]. As part of investigating the anti‐inflammatory activity of the *D. ambrosioides* leaf Aq.E. The ability of Aq.E to inhibit protein denaturation was studied. It turned out that *D. ambrosioides* leaf Aq.E significantly prevented albumin denaturation following thermal stress at physiological pH. According to the results recorded, the ability of the Aq.E to prevent the albumin proteins from denaturation is probably a contributing factor to the anti‐inflammatory activity of *D. ambrosioides* leaves. Given that inflammation is a common response to ischemic conditions, the anti‐inflammatory properties of *D. ambrosioides* leaves, as evidenced by their ability to prevent albumin denaturation, could potentially mitigate the inflammatory response associated with ischemia, thereby offering a protective effect against ischemic damage [57]. The results clearly show that this plant’s leaves possess therapeutic potential as an anti‐inflammatory agent. Considering that ischemia often leads to free radical production and subsequent inflammation, the anti‐inflammatory properties of *D. ambrosioides*, demonstrated by its ability to inhibit protein denaturation, may offer protective benefits in ischemic conditions by reducing inflammation and oxidative stress.

Because the membrane of RBCs is similar to that of lysosomes, cell membrane stabilization is a test commonly employed to investigate anti‐inflammatory effects *in vitro*. One of the limiting processes of the inflammatory response is the stabilization of this membrane, which stops the release of proteases and permits more severe tissue damage. Both H/H have the ability to cause RBC hemolysis [27]. Due to the capacity of *D. ambrosioides* leaves to stabilize membranes, Aq.E has been demonstrated to dose‐dependently suppress hemolysis brought on by both H/H. The anti‐inflammatory properties of *D. ambrosioides* leaves may be the cause of this action.

The anticoagulant effect of *D. ambrosioides* leaf Aq.E was examined utilizing the plasma recalcification assay. The formation of a solid clot with saline took 1.16 ± 0.06 min. Heparin, however, caused the duration to increase to 4.64 ± 0.03 min. It is interesting to note that the time required for clot formation increased as the concentration of *D. ambrosioides* leaf Aq.E. At 40 mg/mL, the strongest anticoagulant activity of *D. ambrosioides* leaf Aq.E was observed, that is, 4.63 ± 0.04 min. Through this study, it was made clear that *D. ambrosioides* leaves present anticoagulant activity. In general, anticoagulants play an important role in the prevention and treatment of thromboembolic disorders. *Dysphania ambrosioides* leaves have demonstrated significant anticoagulant effects, which may be beneficial in ischemic conditions. Ischemia often results from thromboembolic events that block blood flow to tissues, leading to oxygen deprivation and tissue damage. By preventing the formation of blood clots and promoting smoother blood flow, the anticoagulant properties of *D. ambrosioides* could help mitigate the risk of ischemia and its associated complications. Therefore, the therapeutic potential of *D. ambrosioides* as an anticoagulant could be valuable in managing and preventing ischemic events. For a long time, vitamin K‐antagonists, heparins, and their derivatives have been major elements in clinical practice. The deadly side effects of these drugs are well documented in the scientific literature, although their effectiveness remains undisputed. At the same time, the development of a new anticoagulant is very expensive. Thus, a less expensive alternative, less harmful but effective, is the ultimate solution. Currently, several studies focus on powerful anticoagulants of natural origin, and there is an increasing need to consume phytochemicals that have anticoagulant qualities that can lower the risk of coronary heart disease [58].

### 4.3. For the Antioxidant Effect of *D. ambrosioides* Leaf Aq.E Followed IR Injury

For a long time, the involvement of ROS in several cardiovascular diseases has become increasingly evident. In the normal physiological state, the formation of free oxygen radicals is in balance with the quantity. This balance is altered in some physiopathological conditions, such as in the case of ischemic attack followed by reperfusion, explained by the important production of oxygen radicals and/or the decrease in antioxidants, especially during myocardial ischemia. It should be noted that ROS contributes to the lipid peroxidation process, which results in the loss of the integrity, fluidity, and permeability of the membrane. This provides proof that ROS play a part in the etiology of cardiac IR damage [59, 60].

Globally, ischemic heart disease is the primary cause of morbidity and death, primarily due to the severe damage caused to cardiac tissue during the IR sequence [61]. This sequence, while necessary to restore blood flow to preischemic tissue, can paradoxically exacerbate cardiac injury, leading to a reduction in cardiac performance during acute coronary events. The reperfusion phase, though crucial for saving ischemic tissue, can worsen the situation by causing extensive necrosis of irreversibly injured cardiomyocytes [59]. Cellular enlargement, loss of ultrastructural integrity, contraction bands, and the deposition of calcium and phosphate granules are the hallmarks of this injury, leading to intramitochondrial and sarcolemmal damage. These pathological events disrupt ionic flow and fluid regulation, contributing to the overall decline in cardiac function. Therefore, suppressing these myocardial consequences is essential for improving cardiac performance during and after ischemic events [62–64].

The main line of defense against ROS‐induced cardiac tissue damage is the SOD and CAT enzymes, which represent the key radical scavenging phase, thus weakening the IR injury [65]. For lipid peroxidation, MDA is a considerable agent to evaluate the damage of heart tissue caused by free radicals, of which the reduction of the elevation of the level of MDA, and the improvement of the low activities of SOD and CAT are in favor of cardioprotection [55].

In this study, numerous mechanisms were proposed for the explanation of cardioprotection against IR. It is yet unclear how the plant influences the expression of contractile proteins; the capacity for recovery of ROS in the myocardium injured by the plant could be a simple explanation, thus allowing for fewer damaged cardiomyocytes and the preservation of proteins involved in contraction. Indeed, high levels of ROS during the reperfusion phase lead to the death of cardiomyocytes by apoptosis [66]. Our results are consistent with this argument since the ischemic area of the hearts of rats pretreated with the plant Aq.E was lower than that of untreated rats. In addition, the drop in the level of lipid peroxidation following treatment with the plant Aq.E confirms the preservation of cardiac tissue from damage caused by oxidative stress compared with IR groups. The low levels of the scavenger enzymes SOD and CAT in the IR groups were significantly improved in response to the plant Aq.E treatment. Thus, it can be noted that *D. ambrosioides* has a protective activity against the disruption of endogenous antioxidant levels during the IR sequence, through the stabilization of SOD and CAT levels. The attenuation of cardiac lesions induced by IR sequence involves the intervention of antioxidant enzymes, whose activity is regulated depending on cellular needs (since their production can be inhibited or induced by endogenous effectors). Treatment with *D. ambrosioides* Aq.E allowed the elevation of the SOD level, which can lead to the reduction of superoxide (O2‐) in the IR hearts and the improvement of cardiac function. However, this results in an increase of H2O2. In this case, cardiomyocytes generally have two systems to detoxify this peroxide, one of which is CAT, a hemoprotein located in the mitochondria and peroxisomes, involved in the detoxification of H2O2, by converting it into molecular H2O and O2 [56].

Previous phytochemical and computational investigations of *Dysphania ambrosioides* leaves have identified several bioactive compounds that may contribute to its antioxidant and cardioprotective properties. In our earlier study [67], carvone oxide, 5‐isopropenyl‐2‐methylenecyclohexanol, and caryophyllene oxide were identified as the most active molecules present in the leaves. These compounds were predicted to possess notable antioxidant potential, which could partly explain the cardioprotective effects observed in the present study, particularly through the mitigation of oxidative stress associated with myocardial IR injury.

### 4.4. For the Posthemodynamic Parameters of Improvement by *D. ambrosioides* Leaf Aq.E Treatment Followed IR Injury

The sequence of global ischemia (30 min)–reperfusion (120 min) of rat hearts is generally associated with a reduction in contractile function, reflected by an increase in LVEDP. This exacerbates the lesions by causing reduced perfusion in the subendocardial area, which is already heavily compressed during systole. Impaired contractile function is also associated with impaired cardiac inotropism and relaxation mediated by ventricular dysfunction. Reduction of LVEDP following treatment with *D. ambrosioides* leaf Aq.E results in increased blood flow via the ventricular subcardiac region. In ischemic insult conditions, the drop in blood flow to these regions subjected during systole to significant extravascular compression has been well documented, and the Aq.E plant can correct the elevation of LVEDP by restoring normal blood flow to these regions [68, 69].

During the IR sequence, it should be noted that the alteration of cardiac function reflected by ventricular dysfunction is generally caused by an overproduction of ROS [70, 71]. When 30 min of global ischemia and 120 min of reperfusion caused IR injury in the rat heart, similar findings were also noted in this investigation. In the current investigation, arrhythmia and coronary vasoconstriction were noted, along with a notable decrease in LVDP and an increase in LVEDP. A large decrease in the (−) LVdP/dt indicated diastolic dysfunction.

*D. ambrosioides* leaf Aq.E in this study significantly preserved hemodynamic parameters and maintained ventricular function in a dose‐dependent manner, as evidenced by a decrease in preload (LVEDP), an increase in contractility indices (+LVdP/dt), relaxation (‐LVdP/dt), and restoration of LVDP.

## 5. Conclusion

According to the current study, *D. ambrosioides* leaf Aq.E. exhibits strong cardioprotective activity, as evidenced by its ability to mitigate a number of biochemical, hemodynamic, and histological changes brought on by myocardial injury. Its cardioprotective potential is guaranteed by enhanced hemodynamics, restored endogenous antioxidant levels, and improved histopathological features. This broad range of *D. ambrosioides* leaf Aq.E effects lends credence to the idea that using natural products can lessen myocardial IR damage. Therefore, it is possible that the enhanced heart functional recovery following IR injury is due to the antioxidant, anti‐inflammatory, and anticoagulant qualities of *D. ambrosioides* leaf Aq.E.

## Author Contributions

Mounime Kadi contributed to the investigation, methodology development, formal analysis, and preparation of the original draft of the manuscript. Ali Berraouan was involved in the investigation, visualization of the data, validation of the results, and in reviewing and editing the manuscript. Hassane Mekhfi, Abderrahim Ziyyat, and Mohamed Bnouham participated in reviewing and editing the manuscript. Abdelkhaleq Legssyer contributed to the investigation, methodology development, validation of the results, supervision of the work, preparation of the original draft, and reviewing and editing the manuscript.

## Funding

This study was supported by Ministère de l’Education Nationale, de la Formation professionnelle, de l’Enseignement Supérieur et de la Recherche Scientifique.

## Disclosure

All authors have read and approved the final version of the manuscript. Dr Kadi Mouime had full access to all the data in this study and takes full responsibility for the integrity of the data and the accuracy of the data analysis.

## Ethics Statement

The animal experiments conducted in this study were approved by the Institutional Ethics Committee of the faculty of Sciences, Oujda, under the approval number 02/26‐LBBEH‐01, in accordance with the globally recognized norms for the use and care of laboratory animals as stated in the European Community guidelines (EEC Directive of 1986; 86/609/EEC) or the US guidelines (NIH publication #85‐23, revised in 1985).

## Conflicts of Interest

The authors declare no conflicts of interest.

## Data Availability

The data that support the findings of this study are available upon request from the corresponding author.
